# Global Trends in Maternal Mortality and Efforts to Improve Maternal Outcomes Through Sociomedical Interventions

**DOI:** 10.1007/s43032-026-02086-8

**Published:** 2026-05-01

**Authors:** Biraj Sharma, Roger Smith, Craig E Pennell

**Affiliations:** 1 School of Medicine and Public Health, College of Health, Medicine and Wellbeing, The University of Newcastle, University Drive, Callaghan, NSW 2308 Australia; 2 Reproduction and Family Health Program, Hunter Medical Research Institute, Kookaburra Circuit, New Lambton Heights, NSW 2305 Australia; 3 Mothers and Babies Research Centre, The University of Newcastle, University Drive, Callaghan, NSW 2308 Australia

**Keywords:** Maternal deaths, Maternal mortality in low- and middle-income countries, Sustainable Development Goal 3.1, Sociomedical factors

## Abstract

Maternal mortality continues to be a major global health concern that disproportionately affects low- and middle-income countries (LMICs), with the World Health Organisation (WHO) estimating a maternal death occurring every two minutes. The data-sparse LMICs employ a multitude of estimation approaches to gauge maternal mortality ratios (MMR); however, their classification of deaths and reproducibility of estimates remain open to discussion. Despite a considerable reduction of MMR levels since 2000, more recently, the MMR levels in countries including the US have resurged due to the sociomedical crises brought about by the COVID-19 pandemic. The United Nations’ Sustainable Development Goal (SDG) 3.1 aims to achieve global maternal mortality ratios of less than 70 per 100,000 live births and below 140 per 100,000 live births at the national level by 2030. However, recent projections indicate it will remain unmet by a margin of a million maternal deaths. Many LMICs apply the three-delays framework of maternal deaths that requires verbal autopsy to be used in tandem with the identification of maternal deaths. The three-delays model devised in the mid-1990s allows LMICs to gear their resources towards specific intervention points. A significant portion of the existing literature has focused on the description of the magnitude of the issue and the factors precipitating maternal deaths. Innovative solutions have recently been implemented, such as repurposing military helicopters to reduce the delays in managing obstetric complications. Similarly, prospective studies are required to devise ways to address the sociomedical mechanisms underlying maternal deaths.

## Introduction

### Maternal Deaths, Pregnancy-Related Deaths and Severe Acute Maternal Morbidity

Maternal deaths remain stubbornly high in low and middle-income countries (LMIC), while they have become rare events in countries served by well-resourced health systems. Bridging the gap between outcomes in poor and well-resourced settings requires good-quality data to allow measurement of the burden of illness and progress. Multiple measures of maternal death have been developed and used in different settings. These measures include Maternal Death, the Maternal Mortality Ratio, Direct Maternal Deaths, Indirect Maternal Deaths, Pregnancy-Related Deaths and Severe Acute Maternal Morbidity and Preventable Maternal Mortality. Each measure has strengths and limitations.

Maternal death is defined by the WHO as a female death from any cause related to or aggravated by the pregnancy or its management (excluding accidental or incidental causes) during pregnancy and childbirth or within 42 days of termination of pregnancy, irrespective of the duration and site of the pregnancy [1]. Deaths due to obstetric complications and any interventions during pregnancy are classified as direct maternal deaths. In contrast, deaths due to existing health morbidities aggravated by pregnancy are categorised as indirect maternal deaths [2]. In the developing world, the morbidity associated with childbirth may exceed 42 days; hence, reported maternal death rates related to pregnancy may be underestimated [3].

Maternal deaths may also be reported as a ‘pregnancy-related death’, defined as the death of a woman while pregnant or within 42 days of termination of pregnancy, irrespective of the cause of death (obstetric and non-obstetric) [2]. This indicator is generally used in low- and middle-income countries (LMICs) as the accuracy of the cause of death is often uncertain in settings that lack medical verification.

Severe acute maternal morbidity (SAMM), also referred to as a ‘near miss’, is defined as a very ill pregnant or recently delivered woman who would have died had it not been that luck and good care was on her side [4]. Maternal deaths are relatively rare events in well-resourced settings, and SAMM events are more frequent and can, therefore, be used as a more sensitive indicator that allows the development of measures to upgrade obstetric care and improve maternal outcomes.

The Maternal Mortality and Morbidity Review Committee of the Massachusetts Department of Public Health defines Preventable Maternal Mortality (PMM) as ‘the death that may have been averted by one or more changes in the health care system related to clinical care, facility infrastructure, public health infrastructure and/or patient factors.’ [5] All deaths due to haemorrhage and complications from chronic diseases are considered potentially preventable; however, mortality precipitated by pathologies such as amniotic fluid embolism, microangiopathic hemolytic syndrome, and cerebrovascular accidents are considered to be non-preventable. The North Carolina Pregnancy-Related Mortality Review identified improved quality of maternal care as the most important contributor to preventable maternal deaths for the 1995–1999 period.

### The Maternal Mortality Ratios

The maternal mortality ratio (MMR) indicates the risk of maternal death relative to the number of live births. The MMR is expressed as the death of women in the reproductive age group per 100,000 live births in any given year [6]. MMRs are significantly inversely related to national gross domestic product (GDP) with elasticity estimates of US$ 0.36 GDP per capita reduction per year for every maternal death. Kirigia et al., used a double-log econometric model applying the equation: ln(Y) = β₀ + β₁ln(X₁) + β₂ln(X₂) + . + ε, which uses the natural logarithm of both dependent (Gross domestic product ‘Y’) and independent (MMRs ‘X’) variables. This model allowed the investigators to take account of potential non-linearities in the data from the 45 member states in WHO African region used in the study [7].

The WHO has categorised the MMRs of countries as low if less than 100, moderate if 100–299, high if 300–499, very high if 500–999 and extremely high if it exceeds or is equal to 1000 maternal deaths per 100,000 live births [1]. In 2020, three countries, South Sudan, Chad and Nigeria, had MMRs higher than 1000 per 100,000 live births. A lack of access to maternity care was pinpointed as the primary cause for such high figures [8]. MMRs can vary widely within the same country - a recent report on MMRs from Indonesia identified significant variation within its regions; North Sulawesi region was reported to have an MMR of 1811 per 100,000 live births and Java-Bali region had MMR of 95 per 100,000 live births [9].

### Classification of Maternal Deaths and Challenges of Accurately Determining MMRs

Attributing the causes of maternal deaths is crucial in low-resource nations to gear the allocation of resources to improve obstetric outcomes.

**Fig. 1:**
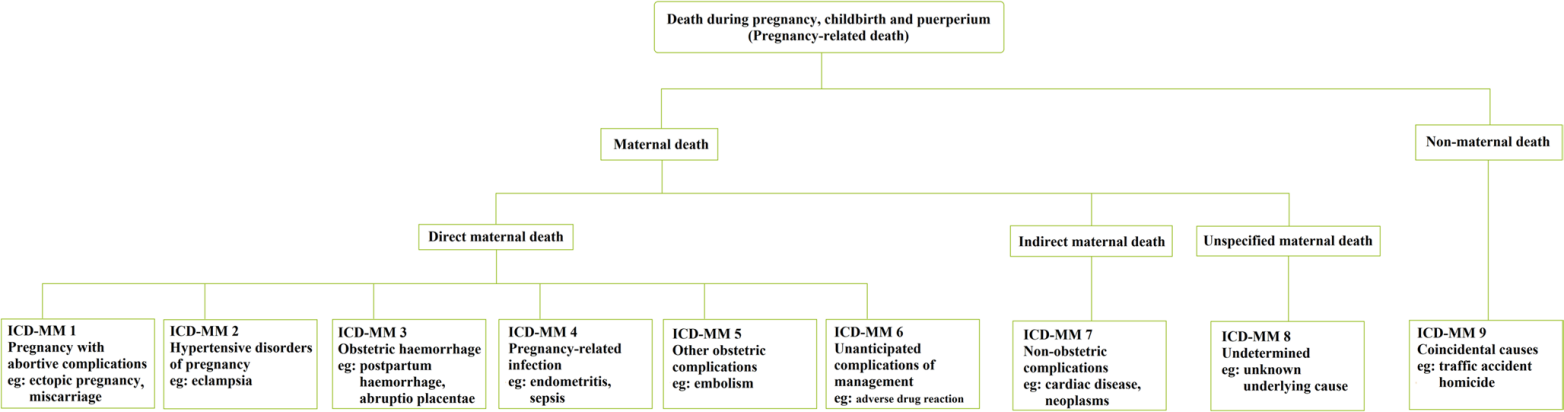
Classification of maternal deaths based on WHO’s application of International Classification of Diseases-10 to deaths during pregnancy, childbirth and puerperium [10, 11]

It can be challenging to identify the etiology of Maternal deaths and discern them from the wider Pregnancy-related deaths. The WHO has endorsed the 10th revision of the International Classification of Disease of Maternal Death (ICD-MM) to help healthcare workers in communities and hospitals categorise maternal deaths [1, 10] (Fig. 1).

The hurdles in accurately identifying maternal deaths are primarily data misclassification and data missingness [1]. For instance, maternal suicides have been a topic of conversation within the obstetric community. Attributing suicide to a particular category within the classification of maternal death is fretted with over-generalisation concerns. Maternal suicides can occur both due to postpartum psychosis as well as pre-existing psychiatric illness; hence the categorisation of suicide remains debatable [12]. Furthermore, to assess accurately cases of maternal deaths in countries with a high prevalence of HIV, the healthcare worker needs to meticulously discern the actual cause of death. The clinical features between the possible clinicopathological causes can overlap, and even a well-trained community health worker or enumerator in surveys may face challenges in categorising the death of a woman. These practical challenges that precipitate data missingness and misclassification at the grassroots compound the obstacles in accurately evaluating MMR levels.

## Global Burden of Maternal Mortality

In 2020, the WHO used a refined maternal mortality estimation model, which employs a Bayesian maternal mortality estimation model (BMat) that extends upon a previous multilevel regression model as well as incorporates an autoregressive integrated moving average (ARIMA) and data adjustments to address data quality (underreporting/misclassification/incompleteness) issues to provide data-driven estimations [13]. The WHO used this approach to estimate 287,000 women died due to maternal causes globally in 2020, which equates to one death every two minutes. The majority of these fatalities occurred in low- and middle-income nations [14]. A woman’s risk of obstetric fatality is as high as one in six in limited-resource countries. In contrast, the risk in northern European nations is one in thirty thousand [15]. The statistics reflect the contrast in maternal care a woman can access in different regions of the world. Most of these maternal deaths have been shown to be preventable, and hence, reducing maternal deaths remains one of the primary goals of public health interventions [16].

Using an ensemble forecasting approach, an approach that relies on a large set of possible combinations of mixed effects models instead of a single one to generate long-term projections [17] created by the Institute of Health Metrics and Evaluation (IHME), the Gates Foundation estimated the global maternal mortality rate to be 152 deaths per 100,000 live births in 2020.

A study of maternal mortality rates across 133 nations showed them to have either stagnated or seen substantial increments during 2016-20. Countries from North America, Western Europe, and the Caribbean reported significant increases using country-level data [8]. In the US, COVID-19 was a contributing factor for up to a quarter of the obstetric deaths in 2020-21, as reported by the US Government Accountability Office [18].

### Maternal Mortality in the Organisation for Economic Co-operation and Development (OECD) Countries

The OECD member nations collectively had an MMR of 15.3 per 100,000 live births as reported by Kurjack et al. in 2021; 98.7% of births in these countries were attended by skilled health personnel [19]. However, even in OECD countries, hurdles exist in generating accurate national statistics; for instance, Canada does not have a national reporting system for maternal mortality, nor does its perinatal surveillance system have access to a comprehensive dataset [20] – highlighting challenges in maternal healthcare improvement even in well-resourced settings.

Within OECD countries MMRs vary with health care systems, ethnicity and socio-economic status. The United States has one of the highest maternal mortality levels among OECD nations [21] in 2020. The MMR of the United States of 17.4 per 100,000 live births was almost three times higher than that of Australia. Within the American women, Black women had an MMR of 37.1 per 100,000 compared to that of 14.7 for white women and 11.8 for Hispanic women [22]. The US is also the only OECD nation that saw its MMR significantly increase over the period of 2000 to 2020 [14]. A recent report by Sharma et al. showed that access to obstetric care services for marginalised women could be improved by introducing targeted interventions. Helicopter retrievals of women from remote mountainous regions in Nepal helped ensure improved maternal outcomes for more than 600 expectant women with obstetric emergencies [23]. Similarly targeted programs for vulnerable women could be applicable in the US. A considerable disparity in MMR existed between Indigenous and non-Indigenous women in Australia. In the 2012–2019 period, MMR in Indigenous women was 17.5 per 100,000 live births and 5.5 per 100,000 live births in non-Indigenous women, as reported by The Australian Institute of Health and Welfare (AIHW) [24]. The report linked the findings to a spectrum of risk factors including difficulty in accessing health services and higher prevalence of substance abuse in the Indigenous community.

Among South Korean primipara women, a population-based retrospective study showed income levels were highly associated with all-cause maternal mortality [25]. A Swiss review observed that women’s risk after a caesarean section had decreased, however, as direct causes of maternal death decreased, death from suicides was becoming a major indirect contributor to MMRs [26]. The maternal country of origin was noted to be a risk factor for obstetric deaths in Spain, with women from South America having had the highest risk [27]. Similar findings of non-Dutch origin women having a higher risk ratio were demonstrated by a study in the Netherlands for the 2006–2018 period, with cardiac complications as the primary contributor to maternal deaths [28]. Haemorrhage remained the most common direct cause of maternal death in OECD countries. However, social parameters such as income strata and country of origin were determinants of MMRs. Indirect causes, such as cardiac complications and mental health disorders, were implicated as major contributors to maternal morbidity and mortality.

### Maternal Mortality in Low- and Middle-Income Countries

In 2017, 94% of all maternal deaths occurred in limited-resource nations as reported by the WHO [1]. Specifically, Sub-Saharan Africa and South Asia accounted for 85% of maternal mortality. In African countries, 75% of maternal deaths were due to post-partum haemorrhage, hypertensive comorbidities, pre-eclampsia and eclampsia in 2020 according to WHO’s integrated African Health Observatory (iAHO) [29].

According to 2023 UN data, reductions in maternal mortality ratios have plateaued during 2016–2020 in 133 countries, the majority of which are low-resource nations [30]. Peters et. from the Urban Institute, Washington D.C, reported that women in low-income nations characteristically have low social standing and often inadequate resources for women’s holistic health [31]. Availability of maternal health services, essential medicines, safe transfusion measures, choice of modern contraceptions, antenatal checkups, healthcare workforce and emergency obstetric services are often not ensured as a basic aspect of healthcare in these nations [29].

## The Three-delay Model for Examining Maternal Mortality

The low maternal mortality in OECD countries indicates that most LMICs maternal deaths are potentially avoidable. Maternal mortality in LMICs has been linked to delays in accessing appropriate care. Delays in seeking care, accessing healthcare providers and effective management of pregnancy complications have been identified under the“three delays for maternal mortality” framework [32, 33]. The three delays refer to the delay in seeking obstetric care when a woman has an obstetric emergency, the delay in transportation to an obstetric care facility, and the delay in receiving appropriate care after the arrival at the health facility. This model recognises the sociomedical barriers to poor maternal outcomes and possible intervention points [34]. More recently, the notion of “it takes a village” to reduce maternal deaths has been put forth by proposition of a 4^th^ delay that emphasises actions the community members can collectively and timely take to ensure favourable obstetric outcomes [35].

### Factors Leading to Delays in Seeking Obstetric Care

Women from LMICs are more likely to depend on other household members to identify pregnancy complications and make decisions on their behalf to seek timely maternal care as reported by a Tanzanian study by Ernest et al. [36]. Risk of delays at home were identified in 29% and 31% of fatalities occurred when decisions to seek care for the complications were made by husbands and mothers, respectively. Underestimating the severity of the risks of maternal complications and poor healthcare system experiences were identified as potential contributors to the delay by Mamady et al. using community and healthcare staff interviews [37]. Barnes-Josiah et al. noted that among Haitian women and their families, level of trust in the quality of obstetric care provided at local clinics and hospitals for pregnancy complications was the determining parameter that lead women to seek medical care [32].

A scoping review of forty-one studies by Hamal et al., deduced that economic constraints, the level of the husband’s education, the presence of older women in the household and women’s exposure to maternal health messages in India were key factors identified in making correct decisions for favourable maternal outcomes [38]. Societies with a culture of early age of marriage and patriarchal customs compound the pitfalls for pregnant women where lives of women are not duly valued [39]. People from these areas are forced to contact traditional healers of their region, which puts the pregnant mother and the baby’s welfare at peril [40]. Traditional birth attendants’ lack of standardised care in these settings magnifies the challenges for expectant mothers and their families.

### Factors Leading to Delays in Transport

Women in remote regions of a country face longer travel times and geographical hazards are particularly significant in areas where motorable roads and railway line connections with urban city centres are not feasible [41]. This is further compounded by the increased cost of transportation. Proper referral systems to specialised centres were inadequate and not timely when the primary point of care could not adequately manage pregnancy complications [32]. In Haiti, it was noted in complicated pregnancies their caretakers often could not manage the referral with the level of acuity required. Further, the complete unavailability of methods of transport has been described in some studies evaluating factors influencing MMR in low-resource countries with up to 28% maternal deaths occurring in these circumstances in the Masvingo region of Zimbabwe [42]. A recent study has shown significant differences in outcomes for preventable deaths of pregnant women in China between rural-urban locales and remote and inland regions with a Risk Ratio of 2.28 times higher in the rural areas compared to urban China in the 2001–2005 period [43]. Inconvenience of transport was identified as one of the chief causes of maternal deaths along with level of health care available. Better allocation of healthcare resources was recommended in the Rio de Janeiro metropolitan region, Brazil, using a Transportation computer-aided design (TransCAD) system – an approach that incorporates both Geographic information system and transportation modelling in a single platform [44]; it was established that the distance travelled to obtain care was a crucial risk factor for pregnancy-related fatalities [45]. Provisions for broader coverage of waiting homes in Brazil were recommended for women approaching term and facing challenges in timely transportation. Conversely, apt repurposing of existing infrastructures for instance, military helicopters to retrieve women in obstetric emergencies in remote regions, has shown potential to help a significant number of these women [23].

### Factors Leading to a Delay in Receiving Care on Arrival at Health Facilities

A substantial proportion of maternal deaths occur in LMICs even after hospital admission [42, 46, 47]. A Malawian study identified prolonged waiting times before receiving care as a factor responsible for higher rates of adverse maternal outcomes [47]. In a Zimbabwean study, sub-optimal training and lack of essential drugs and equipment in the rural health centres were also contributory factors for up to two-thirds of maternal deaths [42]. An absence of guidelines for conditions such as pre-eclampsia, post-partum haemorrhage and manual removal of the placenta has been suggested as a crucial factor contributing to the inability to receive timely obstetric care at health facilities in the Mangochi region of Malawi in 2017 [47].

## United Nations Aspirations – SDG 3.1 and Approaches to Estimating MMRs

The sustainable development goals set up in 2015 by the United Nations General Assembly aim to reduce maternal deaths to 70 per 100,000 live births globally by 2030 and below 140 per 100,000 live births at the national level [48]; however, current rate projections by the WHO and World Bank show that by 2030 the global MMR by 2030 would be 222 per 100,000 live births and SDG 3⸱1 will remain unmet by a million maternal deaths [14].

**Fig. 2:**
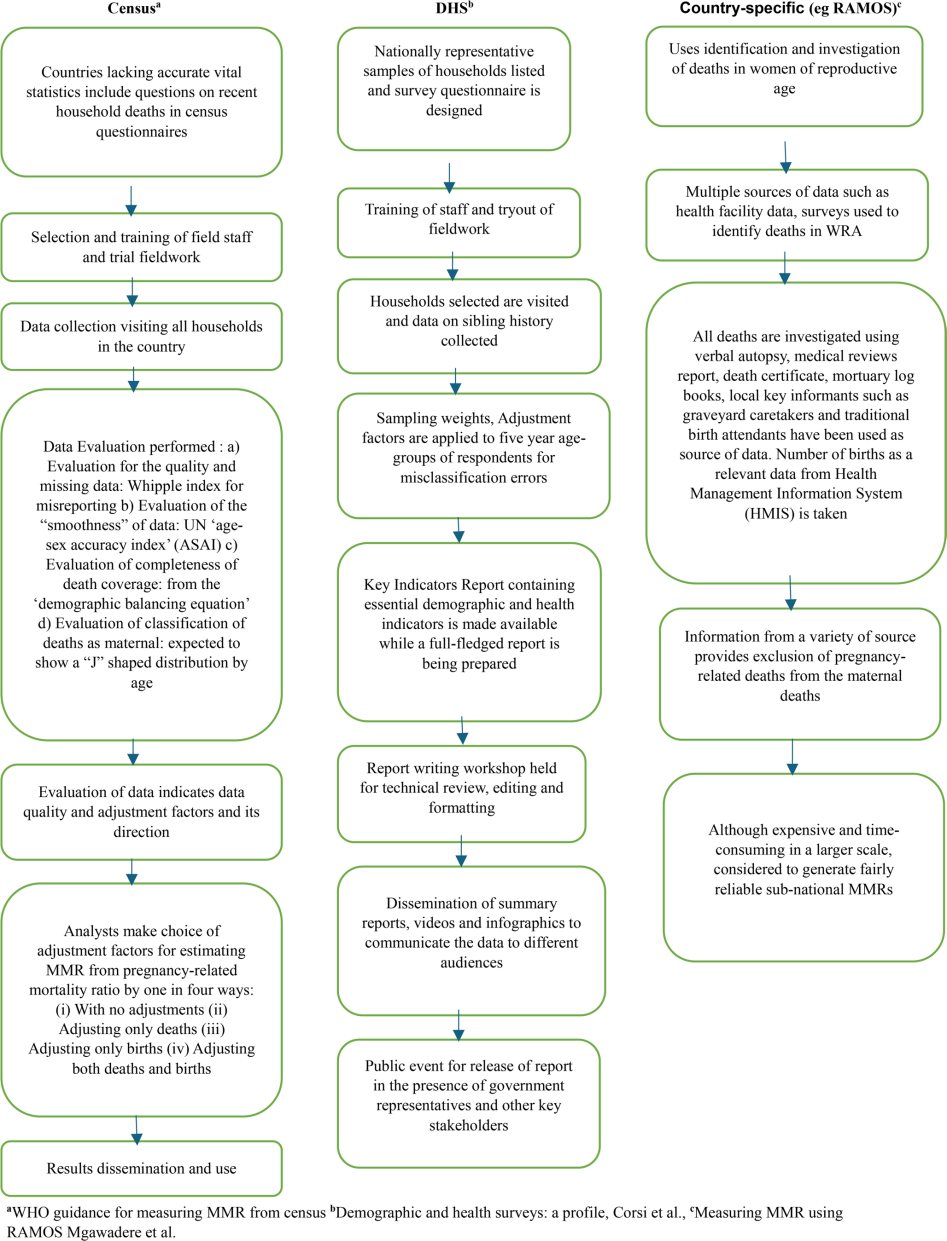
MMR estimation methods used in LMICs. Description: Steps in the estimation of maternal mortality ratios by the census, DHS (Sisterhood) and country-specific (eg. RAMOS) by the low- and middle-income countries

Several data sources form the basis of MMR estimation (Fig. 2). Socioeconomic and geographical parameters significantly impact which data sources are selected in individual countries [49].

1. Civil Registration and Vital Statistics (CRVS) is defined by the United Nations as the continuous, permanent, compulsory, and universal recording of the occurrence and characteristics of important events (live births, deaths, fetal deaths, marriages, and divorces) and other civil status events pertaining to the population as provided by decree, law or regulation, in accordance with the legal requirement in each country [50]. It is the gold standard data source for MMR estimation; however global records indicate that two-thirds of deaths and half of the births are not reported in countries with inadequate systems. CRVS data is unreliable in geographically and economically marginalised settings [51].
2. Sisterhood methods (direct and indirect) are the most used population-based survey approaches. The indirect sisterhood method was initially devised by Graham et al. in the 1980s, and uses questionnaires about surviving and non-surviving female members of families administered by trained healthcare workers [52]. The Demographic and Health Survey (DHS) uses the Direct Sisterhood method proposed by Rutenberg and Sullivan [53]. The direct sisterhood questions are designed to be more extensive than the indirect method. Direct sisterhood questions allow the deaths and births to be placed in a specific chronological timeline, thereby allowing sex and age-specific death rates to be monitored. DHS uses a sample of a small fraction of the population ~ 3000–5000 households deemed nationally representative within the country [54]. The DHS method has potential sources of errors due to the sampling method. In conjunction with the national statistical organisation, the DHS program sampling statistician works out the design for the study sample. The sample is implied to be representative of the national population. However, due to the urban and rural distributions and model insufficiencies likely arising from a nation’s cultural or tribal intricacies, whether a proper representation is achieved in every DHS nation is often uncertain. The DHS sampling manual acknowledges coverage errors and errors during the survey as ‘non-sampling errors’. DHS has a smaller sample size than the census method, affecting the survey’s accuracy. The DHS estimates also depend on the education level of the surviving sisters or siblings, as they are responsible for giving the surveyor an account of the pregnancy status and circumstances of the woman’s death. However, most respondents cannot distinguish between pregnancy-related and maternal mortality from other causes, such as domestic violence and accidental fatalities.
  The sisterhood method was devised to be responded to by a female member of the household, ideally a sister; however, some studies have used sibling histories from male respondents. Both sisterhood approaches yield pregnancy-related death data, but uncertainty exists about the accuracy of the MMR evaluated after data adjustments by this method. The direct sisterhood approach demands additional training resources and supervision compared with the indirect sisterhood method. Over time, with multiple survey waves, estimates generated by the sisterhood approach allow comparable cross-sectional analyses of health intervention indicators and have been used in more than eighty-five nations since 1984 [54].
3. Reproductive age mortality studies (RAMOS): This approach employs active primary and secondary data collection methods involving retrospective and prospective identification of causes of fatalities in women of reproductive age in a defined geographic area or population [55]. RAMOS is conducted in two phases; first, the identification of all deaths of women of reproductive age using health facility data and community surveys; second, all deaths are investigated using medical records, health facility reports and interviews with relatives. The RAMOS approach can provide contemporaneous maternal mortality estimates when a civil registration system is unavailable, with multiple sources used for information on deceased women.
  In a 2008 Philippines study, RAMOS used a standard questionnaire and included rural health units (RHUs), municipal health offices, local civil registries (LCRs), municipal police stations, hospitals, clinics, parishes, and community sources (e.g. village secretaries, relatives of the deceased, non-government organisations [NGOs], and community groups) and identified previously missed maternal deaths at the provincial level [56]. RAMOS studies have been noted to be labour-intensive and uneconomical [57]. The strength of RAMOS, which identifies not only the maternal deaths but also the underlying causes, was a strong factor in it being recommended to evaluate MMR in humanitarian crisis settings such as the Bangladeshi Rohingya refugee camps [58]. Active identification of maternal deaths through systematically administered RAMOS by health workers in the local language in Ghana identified double the number of maternal deaths in the community compared to periods when voluntary reporting occurred [59].
4. Verbal Autopsies (VA): This approach is a questionnaire tool devised by the WHO to analyse the medical history and circumstances leading to the mother’s death using detailed information provided by the respondents. VA interviewers are commonly recruited from local health systems, with the collection of verbal autopsy data considered an ancillary assignment. With assistance from algorithms and computer programs, physicians then analyse the collected data. VAs are often used to verify already identified maternal deaths with standardised VA tools developed in 1994 [60]. It benefits locales where higher expenses have impeded the scope of sampling to obtain an adequate sample size [61]. Verbal autopsy is considered a valuable interim approach for estimating MMR; however, comprehensive and continuation of verbal autopsy programs are recommended in the long term by a 2014 case study of VAs in Mozambique, Bangladesh and Zambia [62]. Other studies have highlighted the large disparity between verbal autopsy and vital registration systems, particularly in settings with a lack of proper training of health workers [63, 64].
  Since 2015, the Bloomberg Data for Health Initiative has facilitated routine application of VA to generate cause of death data in vital statistics systems in developing nations [65]. The informant’s experience and interpretation of key signs and symptoms, the skills and motivation of the interviewers, the type of VA instrument used and the method of analysis of the interview results are all consequential to the accuracy of verbal autopsies [66]. However, empirical evidence suggests that verbal autopsies are labour-intensive. Identification of maternal deaths from verbal autopsy may be challenging given that substantial time and effort are required by the interviewer to reconstruct circumstances prior to death.
5. Population Census: The use of population census data to estimate MMR was initiated by several African countries in the 1970s. The second revision of the United Nations Principles and Recommendations for Housing Censuses (PRPHC) in 2000 included questions on household deaths to address the increased demand to adequately gauge maternal mortality ratios in countries with incomplete vital statistics [67].
  The population census offers an approach to estimate MMR with a marginal expense rather than a large-scale ad hoc survey. This approach is designed to estimate recent maternal mortality by adding a limited number of questions to the population census performed regularly in many countries [68]. Questions regarding the death of women of reproductive age during pregnancy, labour and the postpartum period (six weeks after delivery) are included in the census questionnaires. The census approach provides a pool of observations to allow more detailed interrogation of maternal mortality, for instance, by region or socioeconomic strata. This feature makes for considerable value in planning and implementation of interventions.
  The 2013 ‘WHO guidance for measuring maternal mortality from a census’ provides an overview of the issues in evaluating MMR from a decennial census [55]. The data collection involves field staff selection and training through classroom instruction and trial fieldwork. The guideline also notes that the Ministry of Health should preferably collaborate with the statistics office for effective policy development based on the findings.
  Subsequent to data collection, data evaluation is performed for the quality and missing data by the Whipple index for misreporting and evaluation of the ‘smoothness’ of data by the UN ‘age-sex accuracy index’ (ASAI). The Brass growth balance method, General growth balance method (derived from the ‘demographic balancing equation’), Synthetic extinct generation and Extended synthetic generation are next used to evaluate the completeness of death coverage [55]. Evaluation of classification of deaths as maternal (i.e. deaths occurring during pregnancy, delivery or postpartum period) has no established method but to look at patterns by age group and assess their plausibility as MMR is expected to show a ‘J’ shaped distribution by age, reflecting higher risks at younger and older ages. The analysts will apply upward, or downward adjustment factors predicated on data quality subsequent to the data evaluation to derive the maternal mortality ratios from the pregnancy-related mortality ratios. Thus, the four possible ways of application of adjustment factors are with no adjustments, adjusting only deaths, adjusting only births, or adjusting both deaths and births. For instance, analysts used an adjustment factor of 1.6 for adult female deaths and an adjustment factor of 1.65 for live births in the Lao Democratic Republic in 1995, and the analysts used an adjustment factor of 0.75 for female deaths and an adjustment factor of 1.03 for live births in Paraguay in 2002. The unadjusted MMR for Lao was 821, which was 796 per 100,000 after adjustment. Similarly, the unadjusted MMR for Paraguay was 245, which was 178 per 100,000 live births after adjustment [69].
  The data misclassification and incompleteness have been accounted for by agencies through mathematical models such as the Bayesian model and CODEm [70, 71]. However, the data quality can be improved by strengthening training of enumerators and ensuring an independent verbal autopsy is conducted along with the population based surveys [72].

## Sociomedical Factors Underlying Maternal Mortality in LMICs

Excess maternal mortality is almost invariably a manifestation of social and medical causes within the society. Women often have a poor social standing in LMICs and have little say in the size of their families, with 40–60% of women from Sri Lanka, Egypt, the Dominican Republic, and Jamaica reported to not wanting more children [73]. However, interventions to empower women with the ability to have their desired family sizes through effective contraceptive alternatives have not been accessible. Reaching adequate resources, therefore, remains a substantial challenge for marginalised women.

Reducing maternal mortality rates in marginalised groups of women has been identified as achievable through both Broad and Specific strategies. The broad strategies recommended were increasing the marriage/first birth age and improving family planning, especially for adolescents, to address unsafe abortions [74]. Discouraging child marriages and subsequent early pregnancy ensures the women are physiologically and socially ready to bear and rear children. Providing adolescents safe access to abortion services will prevent morbidities and mortalities from unsafe abortion procedures [75]. The specific strategies identified were establishing antenatal care and particularly early detection of complications [76].

Reducing deaths due to abortion requires a culturally sensitive approach because abortion is taboo across many cultures. Such an approach is particularly beneficial to adolescent and single women who otherwise suffer due to the cultural propensity of minimal access to abortion care [77]. Better care during the post-abortion phase can be ensured through sensitisation of the local communities and the decision-makers’ cooperation towards the women [78].

Women in resource-poor nations have significant challenges in achieving adequate nutrition that aggravate any pre-existing conditions. Associations with severe anaemia and its predictive value for adverse obstetric outcomes have been established chiefly through the increased risks of postpartum haemorrhage [79]. Anaemic women have less capacity to cope with the same volume loss as healthy women due to the reduced capacity to carry oxygen. Availability and aggressive anaemia management through Iron supplements to parturient women have been shown to mitigate morbid outcomes [80]. Violence against women (VAW) affects millions worldwide in physical and psychosocial forms [81]. Compared to their non-abused counterparts, women who have experienced abuse encounter significantly increased pregnancy and neonatal complications. Issues of postpartum depression and difficulty properly breastfeeding the baby are much more likely in abused women [82]. Addressing these factors will be conducive towards reducing maternal mortality through moderating the interplay of these sociomedical factors.

## Future Research on Maternal Mortality in LMICs

A majority of the existing literature on maternal mortality in resource-poor nations has focused on the description of the magnitude of the issue and the factors that precipitate maternal deaths in these settings. The United Nations proposed Sustainable Development Goal 3.1 in 2015 to reduce global maternal mortality to less than 70 per 100,000 live births and, at the national level, MMR less than 140 per 100,000 live births by 2030 – a goal undertaken by all the member nations. Overall, future studies should focus on creative ways to reduce maternal mortality by concurrently tackling the three delays that are associated with excess deaths in LMICs, this approach may also reduce MMRs in wealthier countries, such as the US that have struggled to achieve the low MMRs observed in most members of the OECD.

## Conclusion

Maternal mortality remains a global concern, recently compounded by the far-reaching sociomedical crises associated with the COVID-19 pandemic. Levels of maternal deaths indicate the robustness of national health systems. Herein, we have described the approaches used to document the burden of maternal deaths. Reproducible measures of maternal mortality are required to evaluate the success of interventions to reduce excess mortality. We highlight the value of the three-delays framework for implementing potential interventions to reduce sociomedical mechanisms underlying maternal mortality.
